# Design and Implementation of a Novel Measuring Scheme for Fiber Interferometer Based Sensors

**DOI:** 10.3390/s19194080

**Published:** 2019-09-21

**Authors:** Chao-Tsung Ma, Cheng-Ling Lee, Yan-Wun You

**Affiliations:** 1 Department of Electrical Engineering, CEECS, National United University, Miaoli 36063, Taiwan; 2 Department of Electro-Optical Engineering, National United University, Miaoli 36063, Taiwan; cherry@nuu.edu.tw (C.-L.L.); lovesky791013@gmail.com (Y.-W.Y.)

**Keywords:** optical fiber sensor, fiber interferometer, fast measurement scheme

## Abstract

This paper presents a novel measuring scheme for fiber interferometer (FI) based sensors. With the advantages of being small sizes, having high sensitivity, a simple structure, good durability, being easy to integrate fiber optic communication and having immunity to electromagnetic interference (EMI), FI based sensing devices are suitable for monitoring remote system states or variations in physical parameters. However, the sensing mechanism for the interference spectrum shift of FI based sensors requires expensive equipment, such as a broadband light source (BLS) and an optical spectrum analyzer (OSA). This has strongly handicapped their wide application in practice. To solve this problem, we have, for the first time, proposed a smart measuring scheme, in which a commercial laser diode (LD) and a photodetector (PD) are used to detect the equivalent changes of optical power corresponding to the variation in measuring parameters, and a signal processing system is used to analyze the optical power changes and to determine the spectrum shifts. To demonstrate the proposed scheme, a sensing device on polymer microcavity fiber Fizeau interferometer (PMCFFI) is taken as an example for constructing a measuring system capable of long-distance monitoring of the temperature and relative humidity. In this paper, theoretical analysis and fundamental tests have been carried out. Typical results are presented to verify the feasibility and effectiveness of the proposed measuring scheme, smartly converting the interference spectrum shifts of an FI sensing device into the corresponding variations of voltage signals. With many attractive features, e.g., simplicity, low cost, and reliable remote-monitoring, the proposed scheme is very suitable for practical applications.

## 1. Introduction

In recent years, optical fiber based communication systems featuring low transmission attenuation, high frequency bandwidth, high stability, and immunity to electromagnetic interference, have been widely used in the Internet of Things (IOT) infrastructure, in the big data transmission applications and also the cloud storage systems, in order to meet the increasing bandwidth requirements and security concerns. In addition to its excellent application potentials in the field of data communication, optical fiber is also valued in the field of sensing technology. With proper design, a fiber can be made into a variety of sensing devices because it is highly sensitive to physical parameters such as pressure, temperature, bending, and even refractive index changes. This is because the variation of physical quantities or states will affect the characteristics of light waves transmitted inside the fiber, and thus the physical quantity changes the variations in environmental parameters that can be detected by analyzing the changes of the light wave through the fiber. It is important to note that fiber based sensors integrating optical fiber communication networks can transmit optical signals over long distances and achieve a reliable remote monitoring mechanism.

In the aspects of designing fiber based sensors, there is a huge number of sensing devices that are usually designed based on the theory of interferometers, and the measured phase shifts of the interference waveforms are commonly used to obtain minute changes in the physical parameters of the environment. In fact, the development of feasible fiber optic interferometers with all-fiber configuration has become a very popular research topic recently. These all-fiber interferometers not only have the advantages in measuring precision but also have many superior intrinsic features of optical fibers. For example, optical interference signals do not travel in free space and are not affected by the weather and electromagnetic waves. In the literature, the development of all-fiber interferometers with different configurations has yielded considerable results, such as fiber-optic Michelson interferometers (FMI) [1,2,3,4], fiber-optic Mach–Zehnder interferometers (FMZI) [5,6,7,8], fiber-optic Fabry–Perot interferometers (FFPI) [9,10,11,12,13,14,15,16,17], and fiber-optic Fizeau interferometers (FFI) [18,19]. Typical FI based sensing systems and application examples have been categorized into 13 types, according to the measured parameters, i.e., temperature [20,21], mechanical vibration [22,23], acoustic wave [22,24], ultrasound [25,26], voltage [27,28], magnetic field [29,30], pressure [20,31], strain [21,32], flow velocity [33,34], humidity [35,36], gas [37,38], liquid level [39,40], and the refractive index (RI) [41,42]. Li et al. [20] proposed a cascaded-cavity FFPI to simultaneously sense air pressure and temperature. Huang et al. [21] presented a new sensing mechanism based on a strategically designed micro-cavity FMZI and FFPI for achieving simultaneous and cross-sensitivity free measuring of temperature and strain. The authors of [22] demonstrated that simultaneous measurement of mechanical and acoustic vibration can be realized, using a novel flexible FFPI. Up to 20 kHz acoustic vibration can be measured. In [23], up to 1.5 kHz mechanical vibration was measured using a commercial interrogator and fast Fourier transform algorithms. Low-frequency (around 13 Hz) acoustic pressure was successfully measured by Z. Gong et al. using a simple FFPI [24]. In [25], a high-intensity focused ultrasound (HIFU) was measured by utilizing an ultra-compact, low-temperature crosstalk, two-wave interferometer sensor. W. Zhang et al. [26] designed a miniature FFPI based sensor with spectral sideband filtering for ultrasound and image sensing. Chen et al. [27] used an FMI based sensor to measure voltage and the IEC standard was used to verify the performance of the proposed sensor. In [28], a FFPI driven by electric field forces was able to sense voltage with only 0.1 ms delay. In [29], an AC magnetic field was measured with high sensitivity and correction of temperature crosstalk using an elastic FFPI. A compact magnetic field sensor was designed based on a S-taper and an up-fusion-taper multimodal interference [30]. An absolute pressure sensor was designed based on an external FFPI enclosed in a vacuum cell [31]. Tang et al. [32] designed a dual-tapered photonic crystal fiber (PCF) based FMZI for strain sensing. Zhang et al. [33] proposed an FFPI for low liquid velocity, measured with a high sensitivity of 0.0016 nm/(μL/min). In [34], a hot cavity FFPI based flow sensor with SMF-CDF-SMF structure and with temperature self-calibration was proposed. In [35], a humidity sensor based on a three-fiber, core-offset FMZI was presented. Sensitivities of 0.104 dB/%RH and 0.0272 nm/%RH and 99.61% with correlation coefficients of 99.21% have been demonstrated at 30% and 60% relative humidity (RH), respectively. A humidity sensor designed with a multicore fiber, helical structure, and a gold film reflector was demonstrated by the authors of [36]. Two long period gratings (LPGs) were used to design an ultra-sensitive sensor for measuring underground mine toxic gases such as carbon monoxide (CO) and methane (CH4) [37]. In [38], the CO gas can be effectively detected with high sensitivity using a thin-core FMZI. Liu et al. [39] proposed a temperature-insensitive liquid level sensor based on FFPI with an error less than 0.4% of full scale. Dong et al. [40] used a D-shape fiber modal interferometer to design a liquid level sensor for achieving low temperature cross sensitivity. In [41], a RI sensor with 6.02 × 10^−6^ detection limit and 1.3320 to 1.3465 RIU range was designed with FMZI and Sagnac interferometer. In [42], RI was measured by using an open-microhole FFPI based sensor and Fourier band-pass filtering techniques.

In the above reviewed FI based sensing systems, the design of hardware systems and measuring mechanisms for achieving the desired sensing purposes can be summarized into three combinations: (1) the integration of broadband light source (BLS), optical spectral analyzer (OSA), circulators or power splitters and personal computer (PC) [20,21,30,31,33,35,36,37,40,41]; (2) the integration of an amplified spontaneous emission (ASE) or ASE with tunable optical filter (TOF), photodiode module (PM), data acquisition module (DAQM), circulators or optical couplers (OC) and PC [22,27,38]; (3) the integration of distributed feedback laser (DFBL) or DFBL with the erbium doped fiber amplifier (EDFA), PM or OSA, OC with wavelength-division multiplexer (WDM) module and PC with DAQM [24,25,26,28,29,32,34,39]. The common disadvantage of the existing systems and sensing methods mentioned above include high-cost, as well as being bulky and relatively slow in measuring speed if OSA is used. The above drawbacks have strongly handicapped FI based sensors being widely used in practical applications. To solve this problem, this paper proposes a cost-effective measuring scheme, in which a commercial laser diode (LD) and a photo detector module (PDM) are strategically used to detect the equivalent changes of optical power corresponding to the interference spectrum shifts caused by the variation in measuring parameters. To demonstrate the feasibility of the proposed measuring scheme, a temperature (T) and relative humidity (RH) measurement system based on a polymer microcavity fiber Fizeau interferometer (FMCFFI) [43] is presented, and the related design details are described. The merits of the proposed measuring scheme are low-cost and very easy to be integrated into fiber optic communication systems. In the proposed measurement system, instead of using OSA, a pair of commercial laser diodes (LD) and a photodetector (PD) are used to detect optical power corresponding to the interference spectrum shifts, and an optical/electrical signal processing system with a set of derived algorithms is utilized to convert the spectrum shifts into the equivalent output voltages. In this arrangement, the spectrum scanning time of the OSA can be saved and a low-cost, simple measurement system based on high-sensitivity fiber optic interferometer sensing device can be achieved. To demonstrate the proposed design idea, following this introduction section, the content of the second section reviews the principle and development of a PMCFFI acting as a T and RH sensor. The third section describes the details of the proposed new measuring mechanism for the PMCFFI based T and RH sensing device. A conclusion is then given in the last section.

## 2. Polymer Micro Cavity Fiber Fizeau Interferometer

### 2.1. PMCFFI Manufacturing Steps

**Figure 1:**
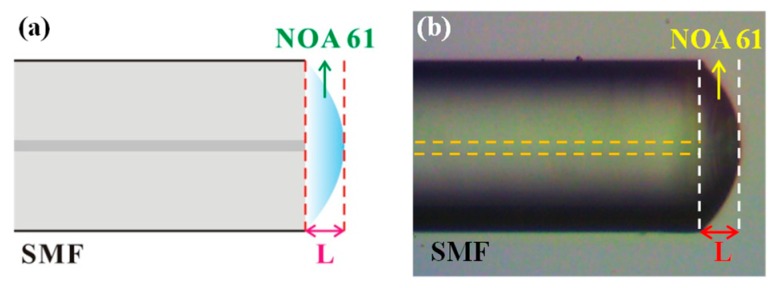
Polymer microcavity fiber Fizeau interferometer (PMCFFI): (**a**) schematic diagram; (**b**) actual photo.

The PMCFFI used in this paper adopts a common single mode fiber (SMF-28), the core diameter of which is 8.2 μm. First, we take a piece of SMF, use a fiber stripper to strip the jacket, wipe it off with an alcohol wipe, and then flatten the end face with a Fujikura fiber cutter CT-30. Next, we drop Norland Products optical glue NOA 61 onto a slide and evenly smooth it to control the thickness of the glue on the SMF end face. After that, we slowly dip the surface of the SMF into NOA 61 and observe it with an optical microscope to make sure that NOA 61 forms a curved surface that serves as the interference cavity. Based on the experiments, we are able to successfully produce many Fizeau interferometer components with different cavity lengths using special dipping techniques. The NOA 61 dipped fiber end face is then exposed by 9.85 W/cm^2^, 320–480 nm UV light. After some time, the liquid monomer molecules are converted into a stable solid polymer resonator. The component is then placed into a 50 °C environment for 12 h to form the required chemical bond between the NOA 61 and the SMF for achieving an optimal bonding. Figure 1a shows the schematic diagram of the PMCFFI, and Figure 1b shows a photo of a PMCFFI component under an optical microscope.

NOA61 is highly hygroscopic and temperature-sensitive; it is a transparent, colorless, liquid monomer that cures under UV light. Through using NOA 61, the pre-mixing, drying or thermal curing typically required in other optical bonding techniques can be avoided, and the curing speed can be extremely fast. It has excellent light transmission, low shrinkage and slight elasticity relative to other optical bonding materials. These characteristics are important to ensure that users can obtain high-quality optical components. In particular, long-term characteristics can be maintained when environmental conditions change. It also has excellent adhesion and solvent resistance after being fully cured by UV light, but it has not yet reached the best adhesion to glass. One week of ageing is required for the chemical bond to be formed between the glass and NOA 61 for optimum adhesion. Alternatively, the best adhesion can be obtained by aging for 12 h at a temperature of 50 °C. When used for glass bonding, NOA 61 can withstand temperature changes from −15 °C to 60 °C before aging. After full aging, it can withstand temperature changes from −150 °C to 125 °C, making it ideal for harsh environment applications.

### 2.2. PMCFFI Interference Principle

**Figure 2:**
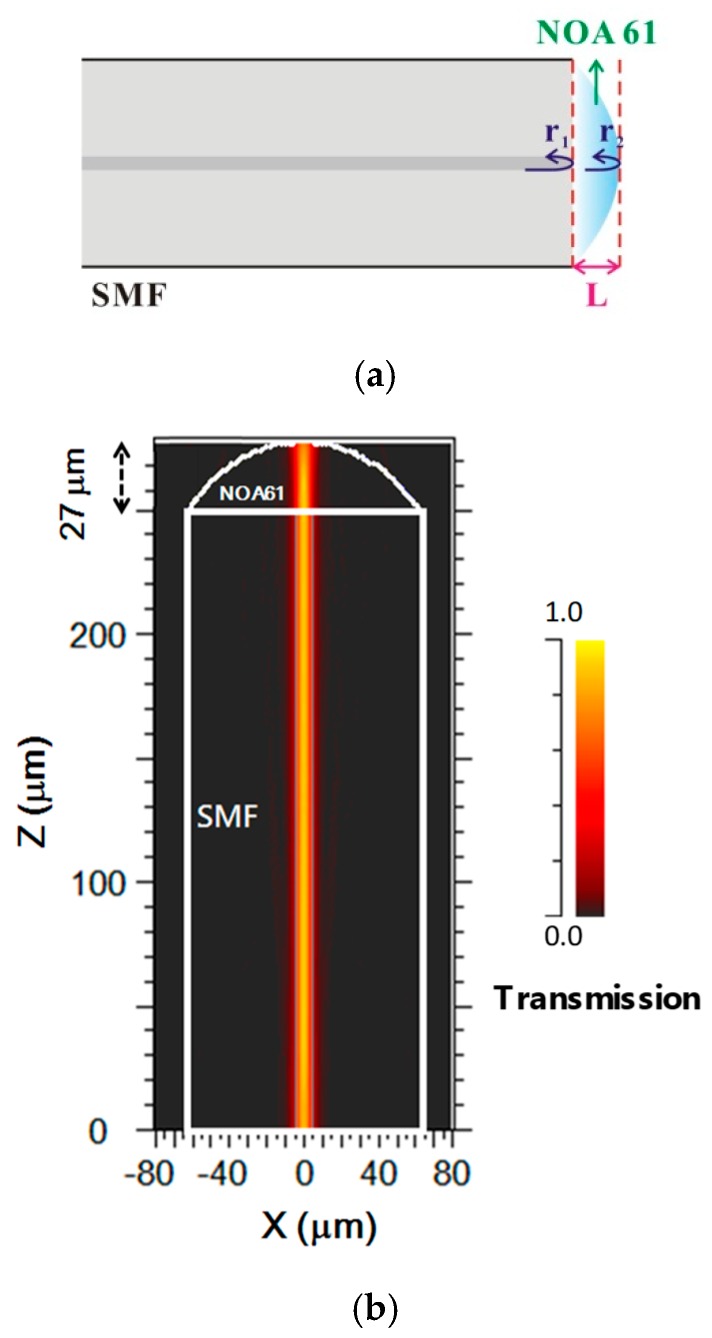
(**a**) Schematic diagram of light propagation characteristics in the resonant cavity of a PMCFFI; (**b**) The transmission of the designed sensing device with the PMCFFI (27 µm).

The PMCFFI adopted in this paper has the advantages of a simple and micro structure, as well as being easy to manufacture, and having a low cost. The main mechanism of its interferometer is described as follows: the light propagates into the SMF, and its mode field distribution is mainly concentrated in the core; when the light reaches the first interface between the SMF and the NOA61 polymer, a portion of the light will reflect first (r_1_), and the other part will be transmitted to the end face of the NOA61 and then reflect (r_2_). Since this configuration adopts two beam interference principles, the reflective surfaces are at the two ends of the polymer. Figure 2a,b shows the conceptual interference diagram and the simulation result of the transmission of the designed sensing device with the PMCFFI (27 μm).

Based on the following simple cavity interference equation, we can derive the sensing characteristics of this PMCFFI. The light travels 2 nL in the NOA 61 polymer resonator, so the cavity phase *δ* is expressed as

(1) δ=(2π/λ)×2nLδ=(2π/λ)×2nL,

where *n* represents refractive index of cavity, and *L* represents resonant cavity length. When *δ* = 2 *mπ*, the interference spectrum shift is at its extremum. As a result, we get Equations (2) and (3):

(2) (2π/λ)×2nL=2mπ;

(3) 2nL=mλ.

In (2) and (3), *m* is an integer. In the interference spectrum, the change of surrounding temperature and relative humidity (*RH*) will cause the cavity length, refractive index, and then spectrum wavelength change, denoting as $L^{\prime }=L+\Delta L$, $n^{\prime }=n+\Delta n$, and $\lambda_{m}^{\prime }=\lambda_{m}+\Delta \lambda_{m}$, respectively. We can, therefore, get Equations (4) and (5):

(4) $$\delta =\left(2\pi /\lambda_{m}^{\prime }\right)\times 2n^{\prime }L^{\prime }=\left(2\pi /\lambda_{m}\right)\times 2nL;$$

(5) $$\Delta \left(nL\right)/nL=\Delta \lambda_{m}/\lambda_{m}$$

Therefore, when the temperature and/or RH rise, the NOA 61 resonant cavity length will increase accordingly. Based on this, the interference spectrum shifts to a longer wavelength (redshift). On the other hand, when the temperature and/or RH drop, the interference spectrum will shift to a shorter wavelength (blueshift). This process proves that the optical path difference changes according to temperature and RH changes, which can be measured by analyzing the interference spectrum shifts.

### 2.3. Experimental Measurement Configuration and Results

**Figure 3:**
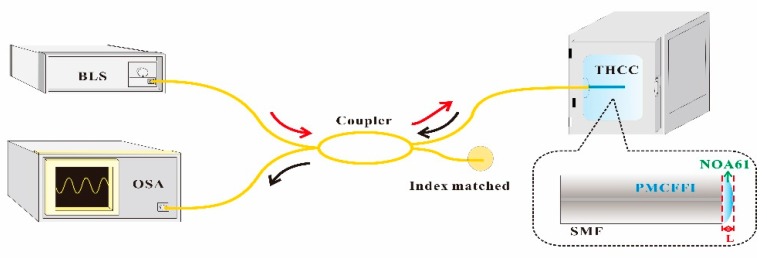
Schematic diagram of the PMCFFI experimental measurement configuration.

**Figure 4:**
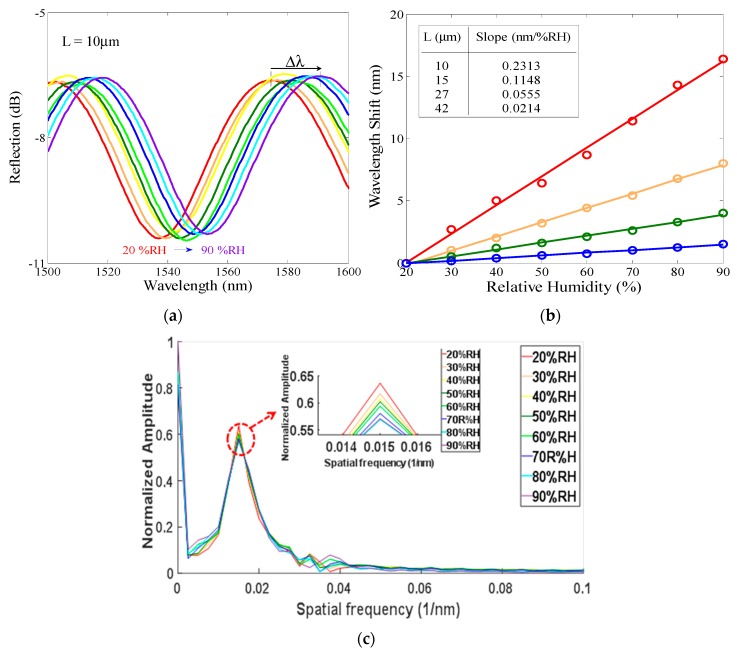
(**a**) Interference spectra of the PMCFFI (L = 10 μm) with only relative humidity (RH) variations; (**b**) Sensitivity comparison of different L based on wavelength shift due to RH changes; (**c**) the FFT result of the PMCFFI (L = 10 μm) with only RH variations (from 20% to 90%).

For deriving a general calculating algorithm for the proposed measuring system, some experimental tests are performed and the related parameters are obtained. The arrangement of experimental measurement is shown in Figure 3. The designed PMCFFI is placed in a temperature/humidity controlled chamber (THCC). A BLS is used as the signal source and connected to the PMCFFI through a set of 2 to 2 optical couplers. When the light passes through the end face of the PMCFFI, the reflected light will return to the optical coupler and then enter an OSA for interference spectrum analysis. Here, the cavity lengths of PMCFFI are developed at 10, 15, 27 and 42 μm, respectively. Figure 4a shows the interference spectra of L = 10 μm PMCFFI with only RH variations. A complete set of RH sensing results with these components is obtained and found to have a linear response in phase shift, as shown in Figure 4b. As can be seen, different sensitivities are observed in different cavity lengths. To observe the free spectral range (FSR) of the measured interference, a set of FFT results of the PMCFFI (L = 10 μm) with only RH variations (from 20% to 90%) are shown in Figure 4c.

## 3. Development of the Proposed Measurement System Based on PMCFFI Sensing Device

### 3.1. Measurement Principle and Implementation

**Figure 5:**
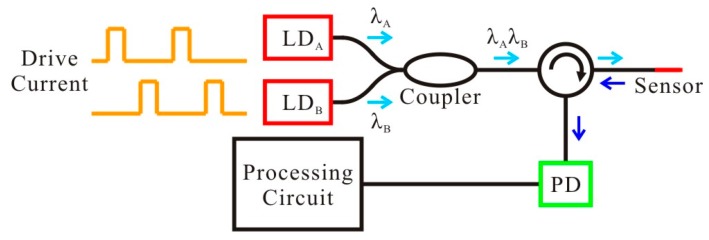
System configuration of the proposed fast measurement system with a PMCFFI sensor.

As addressed in the previous section, the output signals of the proposed PMCFFI sensing device are interference spectrum shifts, or equivalently, the variations in optical power at a specified wavelength. With this concept in mind, one can use a laser diode (LD) with the desired wavelength and a photodetector (PD) acting as the signal detector together with a general fiber network to construct a simple and cost effective measuring system. In practice, through a signal processing circuit, the measured optical power signals (dBm) can be converted into electrical signals (mV) and input to a computer for spectrum phase shift calculation and displaying the measuring RH. The structure and operating mechanism of the proposed measurement system are shown in Figure 5.

**Figure 6:**
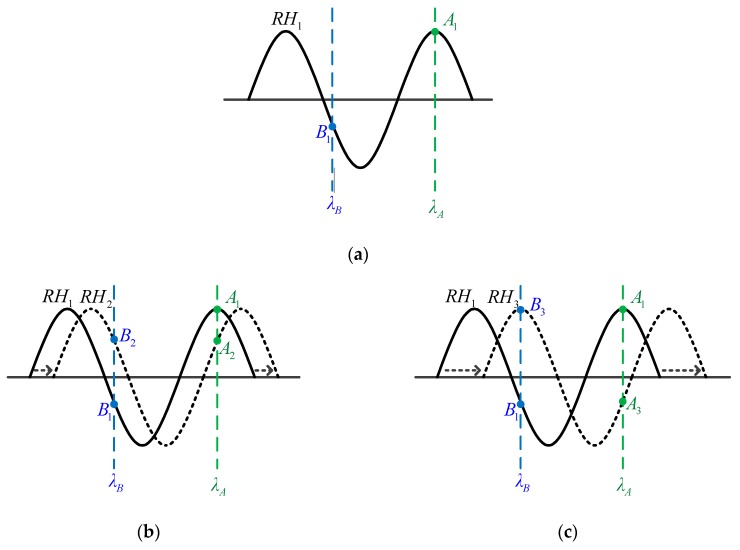
Schematic diagrams of spectrum shift monitoring on two wavelengths: (**a**) initial state; (**b**) right shift within feasible detecting range; (**c**) when the spectrum shift has reached its extremum.

To have a clear picture of the proposed measuring concepts, Figure 6 shows the schematic diagrams of spectrum shift monitoring at two wavelengths. First, the interference spectrum (usually a sine-like or cosine-like periodic function) of the PMCFFI sensing device at a given temperature (20 °C) is first measured using OSA. Next, a certain period in the interference spectrum near the optical communication band is selected for sensing. In this case, a pair of LDs with wavelengths of λ_A_ and λ_B_ are used to monitor reflective optical powers of the two wavelengths on the spectrum, as shown in Figure 6a. The change of the sensed parameters will cause interference spectrum shifts. By monitoring the optical powers (dBm or voltage values in mV) at λ_A_ and λ_B_, the interference spectrum shifts can be precisely calculated, and thus the changes in the monitored parameters can be obtained. Since the reflective power spectrum is approximate to a sine wave, we can obtain better linearity within the range of around π/2; this should be taken into consideration when selecting the wavelength of LDs, so that the selected LDs can monitor, with better linearity, during the different states of spectrum phase shifts. To determine if the spectrum shift exceeds the feasible detecting range in a certain design (theoretically less than π), we can observe the reflective power change of the two wavelengths: if the two power variation polarities remain opposite to each other, the spectrum shift has not reached its extremum, as shown in Figure 6b. If the two power variation polarities become the same, the spectrum shift has reached its extremum, as shown in Figure 6c. After the test is done, a microprocessor with the derived calculating algorithms, along with some signal processing circuits, are integrated to complete the hardware prototype of the proposed measuring system.

### 3.2. System Prototype

**Figure 7:**
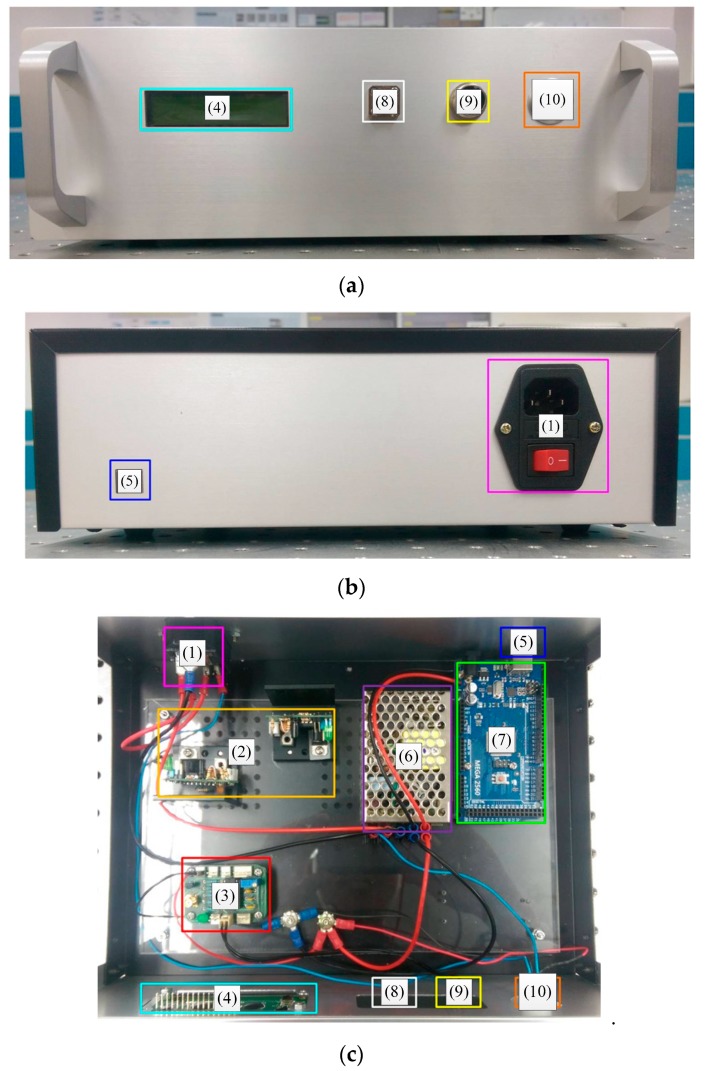

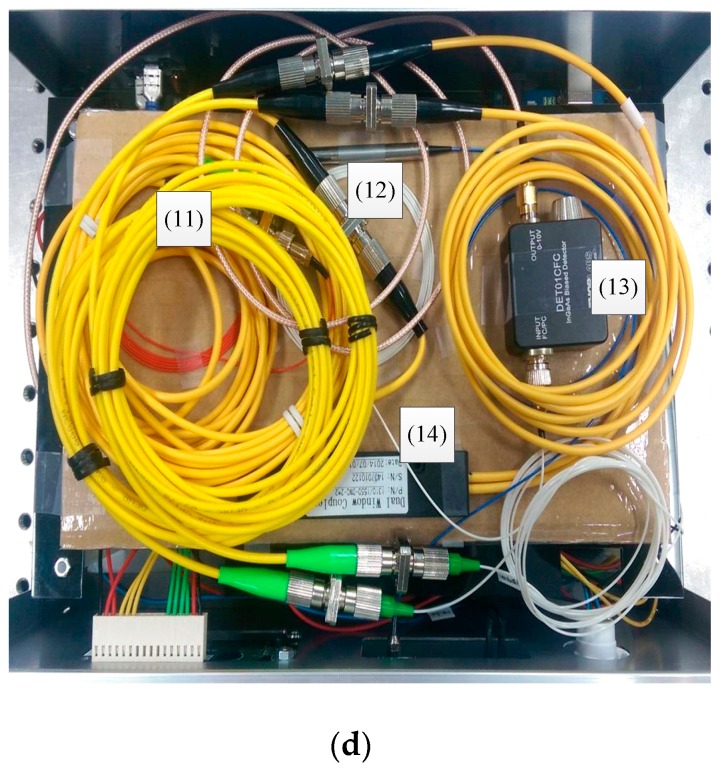
The developed prototype: (**a**) front view; (**b**) back view; (**c**) lower layer; (**d**) upper layer.

Based on the theoretical analysis and the required sensing of the displaying functions, we have designed and practically constructed the hardware system as shown in Figure 7, where we can see (1) is the power supply port and the main power switch, (2) is a dc power control module, (3) is a voltage converting circuit for the PD and the thermistor, (4) is a liquid crystal display (LCD), (5) is a USB port, (6) is a 110V_AC_/12V_DC_ adapter, (7) is the Arduino Mega 2560 control board, (8) is a FC/FC adaptor, (9) is the thermistor sensing port, and (10) is the power switch. In Figure 7d, we can see that the upper layer contains (11) fiber pigtail, (12) optical circulator, (13) photodetector (Thorlabs DET01CFC), and (14) the optic coupler. The two ports on one end of the 2 to 2 optical couplers are connected to the FC/APC, and the other two ports are connected to optical circulator inputs. The two optic circulator outputs are connected to the proposed PMCFFI device and the PD input, respectively.

### 3.3. Measurement Results

**Figure 8:**
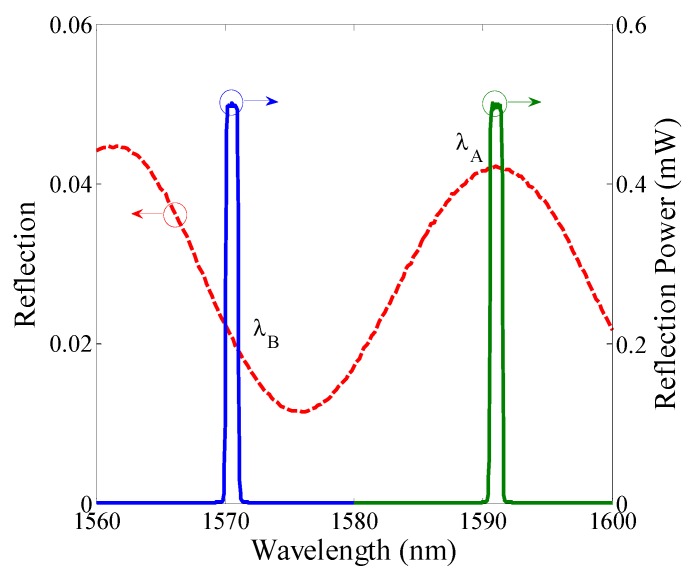
Using 2 laser diodes (LDs) to monitor the reflective power spectrum at 20 °C.

After the measuring system is completed, the initial spectrum of the PMCFFI with L = 27 μm is firstly measured by the OSA. The two LD monitoring wavelengths are then selected at λ_B_ = 1571 nm and λ_A_ = 1591 nm, as shown in Figure 8.

As shown in s we can have the following equations:

*V*_1_ = *a*(*RH*_0_ − *b*_1_) (6)

**Figure 9:**
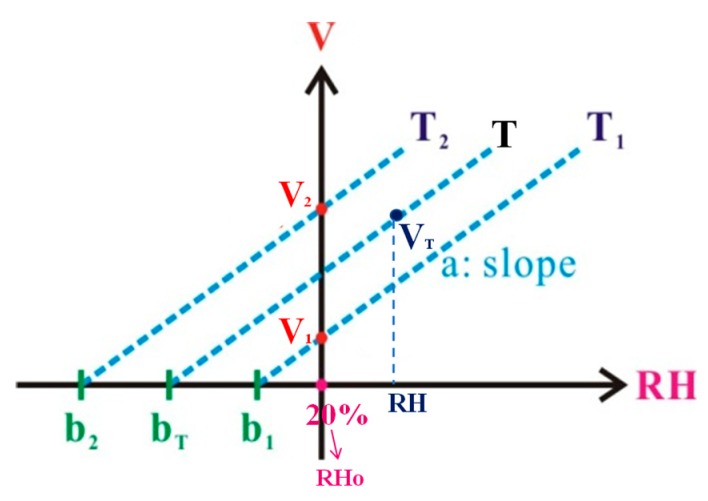
Relationship between voltage and RH at different temperatures.

*V*_2_ = *a*(*RH*_0_ − *b*_2_) (7)

where *V_1_*/*V_2_* represent the measured voltage values at initial *RH_0_* = 20% and for *T_1_*/*T_2_* respectively, and *a* represents the voltage-RH slope, *b_1_*/*b_2_* represent the two x-intercepts for *T_1_*/*T_2_* respectively. To derive a general equation describing the relationships among the voltage, *V*, *T* and *RH*, the following equation can be obtained from Figure 9:

*V*_T_ = *a*(*RH* − *b*_T_) (8)

Based on the geometric relationship shown in Figure 9, *b_T_* can be derived as follows:

*b*_T_ = *b*_1_ + (*b*_2_ − *b*_1_)(*T − T*_1_)/(*T*_2_ − *T*_1_) (9)

Using Equations (6), (7) and (9), b_T_ can be rewritten as:

*b*_T_ = (*RH*_0_ − *V*_1_/*a*) + (−*V*_2_/*a* + *V*_1_/*a*)(*T* − *T*_1_)/(*T*_2_ − *T*_1_) (10)

Substituting Equations (10) into (8) gives the following relationship:

*V*_T_ = *a*(*RH* − *RH*_0_) + *V*_1_ + (*V*_2_ − *V*_1_)(*T* − *T*_1_)/(*T*_2_ − *T*_1_) (11)

For a given operating temperature range (*T_1_*~*T*_2_), the related parameters, *a*, *RH*_0_, *V*_2_, *V*_1_, *T*_1_ and T2 can be decided by performing some initial experiment tests. It follows that the monitored RH value can be achieved and shown in a calibrated voltage *(V*_T_*)* for any temperature between *T*_2_ and *T*_1_.

**Figure 10:**
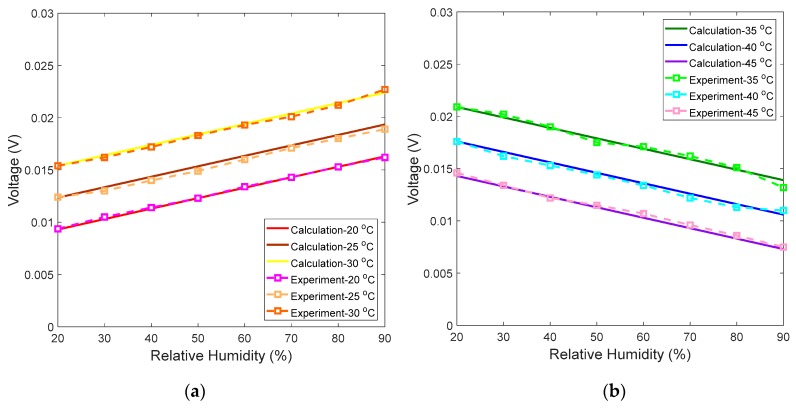
Comparison of calculated and experiment results of voltages vs. RHs at (**a**) 20 °C, 25 °C, and 30 °C, with λ_B_; (**b**) voltages vs. RHs at 35 °C, 40 °C, and 45 °C, with λ_A_.

**Figure 11:**
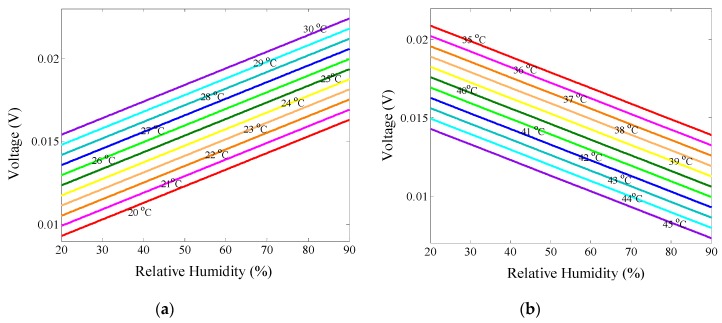
The detailed results of the measured voltages vs. RHs: (**a**) 20 °C to 30 °C, with λ_B_; (**b**) 35 °C to 45 °C, with λ_A_.

To test the performance of the proposed measuring system, the PMCFFI with 27 μm cavity is chosen for some practical measurements. First, the temperature is fixed at 20 °C, and the RH varies from 20% to 90%. It should be noted that the NOA61’s temperature operating range is from –150 °C to 125 °C; however, for demonstration purposes the temperature range of 20 °C to 45 °C is tested in this study. In order to maintain good monitoring linearity, we observe optic power of λ_B_ for temperatures ranging from 20 °C to 30 °C; for the temperature range of 35 °C to 45 °C, we observe optic power of λ_A_. Figure 10a shows the calculated and experimental values at 20, 25, and 30 °C, and Figure 10b shows the calculated and experimental values at 35, 40 and 45 °C. As can be observed, the experimental results are very close to calculation results. Finally, the experimental values of *V_1_*, *V_2_*, and the calculated *a* are used in the derived voltage calculating Equation (11) and written into the Arduino Mega 2560 control board. This completes parameter setting of the measuring system for the chosen PMCFFI sensing device. In this study, a thermistor is used to measure the operating temperature (*T*) in order to correctly determine the voltages corresponding to the monitored RHs. Figure 11 shows a set of detailed measurement results.

## 4. Conclusions

This paper has presented a novel measuring scheme for a PMCFFI based fast and sensitive temperature and RH measurement system, suitable for long-distance monitoring system states or environmental parameters. The measurement system eliminates expensive devices, such as BLS and OSA, that are normally required to measure the interference spectrum shifts of fiber interferometers. In the proposed measuring mechanism, with the derived Ts, RHs and voltages converting algorithms, commercially available LD and PD can be used to measure optical power, and an optical/electrical signal processing unit has been adopted to convert the optical powers, corresponding to a certain spectrum shifts, into the equivalent voltages. It is worthwhile noting that, although the aging effect with regard to long term laser exposure is not obvious in the designed PMCFFI, the laser power should be optimally regulated to further minimize the possible aging effect in long-term operations. The authors believed that using the proposed measuring scheme, most of the optical fiber sensors on FI developed in the literature can be easily implemented, and that a low-cost, reliable and easy-to-use measurement system can be developed for a variety of practical applications. It is also reasonable to note that the proposed sensing mechanism has significant commercial potential.
